# A Bayesian Model Averaging Approach to the Quantification of Overlapping Peptides in an MALDI-TOF Mass Spectrum

**DOI:** 10.1155/2011/928391

**Published:** 2011-05-23

**Authors:** Qi Zhu, Adetayo Kasim, Dirk Valkenborg, Tomasz Burzykowski

**Affiliations:** ^1^Department of Electrical Engineering, ESAT/SCD Katholieke Universiteit Leuven, Kasteelpark Arenberg 10, Bus 2446, 3001 Heverlee, Belgium; ^2^Wolfson Research Institute, Durham University, Queen's Campus University Boulevard, Thornaby, Stockton-on-Tees TS17 6BH, UK; ^3^Flemish Institute for Technological Research (VITO), Boeretang 200, 2400 Mol, Belgium; ^4^I-BIOSTAT, Hasselt University, Agoralaan, Building D, 3590 Diepenbeek, Belgium

## Abstract

In a high-resolution MALDI-TOF mass spectrum, a peptide produces multiple peaks, corresponding to the isotopic variants of the molecules. An overlap occurs when two peptides appear in the vicinity of the mass coordinate, resulting in the difficulty of quantifying the relative abundance and the exact masses of these peptides. To address the problem, two factors need to be considered: (1) the variability pertaining to the abundances of the isotopic variants (2) extra information content needed to supplement the information contained in data. We propose a Bayesian model for the incorporation of prior information. Such information exists, for example, for the distribution of the masses of peptides and the abundances of the isotopic variants. The model we develop allows for the correct estimation of the parameters of interest. The validity of the modeling approach is verified by a real-life case study from a controlled mass spectrometry experiment and by a simulation study.

## 1. Introduction

Peptide-centric techniques are gaining a lot of interest for the search of new protein biomarkers, surrogate endpoints, or markers for classification of diseases. Typically, such techniques extensively use mass spectrometry (MS) for protein-expression profiling, because they promote the high-throughput quantitative characterization of a proteome. MS allows to separate peptides, present in a sample, according to their masses. It also provides a measure of abundance of the peptides. By comparing the protein abundances for different samples, differentially expressed proteins can be found. By analyzing the proteins, important information about, for example, mechanisms of disease can be obtained.

In this paper, we consider the problem of the quantification of overlapping peptides in a high-resolution matrix-assisted laser desorption and ionisation/time-of-flight (MALDI-TOF) mass spectrum (MS).

Peptides are chains of amino acids and are composed of atoms of five chemical elements: carbon (C), hydrogen (H), nitrogen (N), oxygen (O), and sulphur (S). Because the chemical elements have different isotopes, peptides can have different isotopic variants, which differ with respect to their weights. For a peptide of a known chemical composition, the probability of occurrence of these variants is called the* isotopic distribution*. It follows that, in a singly charged high-resolution mass spectrum, a peptide produces a series of peaks that are separated by one mass unit (dalton, Da) and that correspond to different isotopic variants of the peptide. These peaks are called * isotopic peaks*. Their relative heights pertain to the probabilities of the isotopic distribution of the peptide.

**Figure 1:**
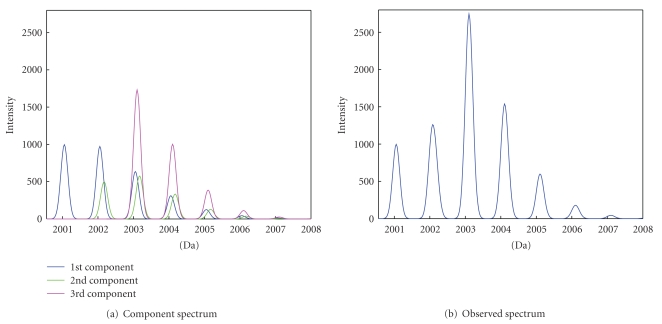
The observed spectrum (b) and its corresponding true underlying peptide components (a).

A “cluster” of peaks observed in a mass spectrum can be produced by more than one peptide. This happens if two peptides differ in mass by at most a few mass units. Such peptides are called * overlapping peptides*.  Figure 1 illustrates a possible scenario for the case of no measurement noise. It shows, in Figure 1(a), isotopic peaks for three overlapping peptides. The resulting observed * joint spectrum* is presented in Figure 1(b), with a “cluster” of superimposed peptide peaks. Our key interest is to quantify the true underlying peptides, as displayed in Figure 1(a). The quantification means a proper assessment of (1) the number of overlapping peptides (components), (2) the * monoisotopic masses* of the peptides, that is, the masses of the isotopic variants that contain the most abundant isotopes of chemical elements constructing the peptides, and (3) the corresponding abundances of the peptides.

Several approaches to the problem of quantification of overlapping peptides have been proposed. Schulz-Trieglaff et al. [1] and Lange et al. [2] developed a peak-picking algorithm by means of a wavelet function, combined with a greedy search to quantify the overlapping peptides. This method has two limitations: (1) often no unique solution can be found for the wavelet functions to fit to the peptide profiles, and (2) greedy search is often problematic in that it can either include noise peaks as peptide peaks or discard peptide peaks, depending on the fit to the wavelet functions. These limitations acting together can lead to nonidentification or misidentification of the overlapping peptides. Breen et al. [3] suggested to model the isotopic distribution by a Poisson approximation, which can also be used to identify overlapping peptides. The method is based on the summary statistics of the original data. This limits the application of the method, for example, by not allowing for the estimation of the mass locations of these peptides. Moreover, the use of the summary statistics could result in severe inefficiency of the parameter estimates. Especially when there is discrepancy between the true isotopic distribution and the Poisson-approximated one, it would incur biased quantification for the parameters of interest, for example, the relative abundance(s) of these peptides.

The quantification of the overlapping peptides is a difficult problem, because the data may contain a very limited amount of information that can be used to distinguish between different configurations of the number, location, and abundances of the peptides, which might have led to the observed joint spectrum. A possible solution is to use prior information that could increase the information content of the data. To this aim, in the paper we propose to use a Bayesian model to analyze high-resolution mass spectra with overlapping peptides. The model allows to use the prior information about, for example, the possible location of the peptides and about their isotopic distribution. It is important to note that the prior information reflects a prior knowledge that can be helpful in analyzing the data but does not necessarily need to exactly represent the data. In other words, the prior and the data can come from different types of proteins. This is because, in the Bayesian framework, the posterior is (combined) information of the prior and the data, being closer to the source that contains more information.

Our paper is organized as follows.  Section 2 presents the shape representation of the MS data that we will consider for our modeling. In Section 3, we discuss the prior information that can be used in analyzing MS data with overlapping peptides. The details of the Bayesian modeling approach are formulated in Section 4. In Section 5, results of an analysis of real-life data sets and a simulation study are presented. Finally, concluding remarks are given in Section 6. 

## 2. Shape Representation of a Mass Spectrum

For the data representation, used for the modeling approach, one way is to base the analysis on the summarized information of the MS data by, for example, using only one data point representing one observed peak. In principle, this implies a severe information reduction and may consequently cause biased estimation. In order to avoid the problem and retain all the information from the data, we consider the original setting of the MS data by means of the (peak-)shape representation.

To work with the shape representation, all mass coordinates and their corresponding intensities are considered. Assume that, for a peak cluster observed in a mass spectrum, as shown in Figure 1(b), we have got *N* intensity measurements, denoted by *y*_*j*_ (*j* = 1,…, *N*), and obtained at masses *x*_*j*_. The intensity at mass coordinate *x*_*j*_ is a sum of intensity measurements of all the isotopic peaks of the peptides that are present at that coordinate. Thus, for the example shown in Figure 1, *y*_*j*_ = *h*_1_ *ψ*(*x*_*j*_; *μ*_1_, *σ*_*s*_^2^) + *h*_2_ *ψ*(*x*_*j*_; *μ*_2_, *σ*_*s*_^2^) + *h*_3_ *ψ*(*x*_*j*_; *μ*_3_, *σ*_*s*_^2^), where *ψ*(*x*; *μ*, *σ*_*s*_^2^) is a suitable function capturing the shape of the peak envelope like, for example, the normal function (either cdf or pdf) with *μ*_*q*_ and *h*_*q*_ denoting, respectively, a mass location and an overall abundance parameter for the *q*th overlapping peptide, and *σ*_*s*_, a dispersion parameter.

The shape representation uses the full content of the MS data and therefore, in principle, allows for a more efficient inference. 

## 3. Available Prior Information

As mentioned in Section 1, the use of prior information could increase the information content of the MS data and allow for a more efficient quantification of overlapping peptides. Such information is indeed available. 

### 3.1. Prior Information for Monoisotopic Mass

**Figure 2:**
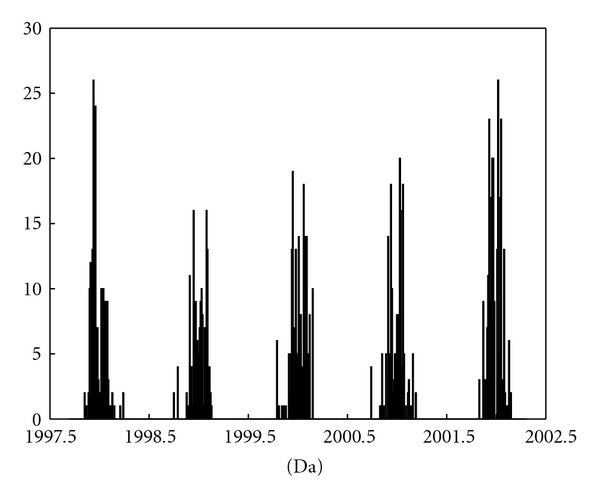
Histogram of the monoisotopic mass locations of peptides in the mass range of 1997.5–2002.5 Da in the NCBI data set.

The RefSeq database of the NCBI, available at http://www.ncbi.nlm.nih.gov/RefSeq/, provides the monoisotopic masses of human peptides. When accessed on February 27, 2008, for the human proteome, the database contained amino acid sequences for 132,292 proteins. Performing an *in silico* digest by trypsine resulted in 2,616,371 peptides with monoisotopic masses between 400 and 4000 Da, with 306,427 unique atomic compositions.  Figure 2 presents the number of peptides with monoisotopic masses appearing in small intervals of 0.01 Da around the mass range of 2000 Da. It can be observed that the monoisotopic masses vary around integer values. Moreover, there are regions where no peptides can be found. This prior information can be quantified by using an appropriate prior distribution in modelling MS data. 

### 3.2. Prior Information for the Isotopic Distribution

The NCBI data can also be used to extract information about possible forms of the isotopic distribution of peptides. Note that, to compute the isotopic distribution, information about the chemical composition of the peptide is needed. Such information is not available in a mass spectrum. However, one can predict the distribution by considering the variability of the distribution of peptides with a similar monoisotopic mass.

To this aim, the isotopic distribution can be modeled by using a polynomial model, with the monoisotopic mass as a covariate [4, 5]. Valkenborg et al. [5] suggested that a fourth-order polynomial is sufficient for modeling purposes. The model is applied to the * isotopic ratios*, which are defined as follows. Let *p*_1_, *p*_2_, *p*_3_, and so forth denote the probability of occurrence of, respectively, the first (monoisotopic), second, third, and so forth (with respect to the increasing mass), isotopic variant of a peptide, given by the isotopic distribution. The * common reference ratios* are defined as follows: *R*_1_ = *p*_1_/*p*_1_ = 1, *R*_2_ = *p*_2_/*p*_1_, and so forth. Thus, they give the probability of occurrence of an isotopic variant relative to the probability of the monoisotopic variant. The * consecutive isotopic ratios* are defined as follows: *C*_1_ = *p*_1_/*p*_1_, *C*_2_ = *p*_2_/*p*_1_, *C*_3_ = *p*_3_/*p*_2_, and so forth. Thus, they give the probability of occurrence of an isotopic variant relative to the previous variant. Note that the two sets of ratios are equivalent, because *R*_*l*_ = *C*_1_ *C*_2_ ⋯ *C*_*l*_.

We used the approach by fitting the following model to the logarithms of the consecutive ratios of the isotopic distributions of peptides from the NCBI data set:

(1) $$\begin{array}{c} \log\;C_{l}=\overset{4}{\underset{k=0}{\sum }}\beta_{k}{\left(\frac{m}{1000}\right)}^{k}+ɛ, \end{array}$$

where *m* is the monoisotopic mass of the peptide and *ɛ* ~ *N*(0, *σ*_*l*_^2^). Model (1) was applied to the logarithms of ratios *C*_*l*_, and not to ratios *R*_*l*_, because the assumptions of the model were more appropriate for the former set of ratios.

The estimated coefficients of the model for isotopic ratios *l* = 2 to 8 are shown in Table 1. They allow to infer the form of the isotopic distribution of a peptide with monoisotopic mass *m*. Again, this prior information can be quantified by using an appropriate prior distribution in modelling MS data.

#### 3.2.1. Reparameterization as A Virtual Constraint of the Isotopic Ratio Estimates

Due to the fact that *R*_*l*_ = *C*_1_ *C*_2_ ⋯ *C*_*l*_, the overestimation of consecutive ratio *C*_1_ would result in the over-estimation of all the common reference ratios *R*_1_ to *R*_*l*_. To circumvent the problem, the ratios can be reparameterized. Recall that *p*_*l*_ is the probability of occurrence of the *l*the isotopic variant and thus ∑_*i*=1_^*L*^ *p*_*l*_ = 1, where *L* is the total number of isotopic variants, observed for a peptide. As *R*_*l*_ = *p*_*l*_/*p*_1_, we then have

(2) $$\begin{array}{c} R_{2}=\overset{L}{\underset{l=2}{\sum }}R_{l}-\overset{L}{\underset{l=3}{\sum }}R_{l}=\frac{1-p_{1}}{p_{1}}-\overset{L}{\underset{l=3}{\sum }}R_{l}. \end{array}$$

Hence, instead of putting a prior on *R*_2_, we use the equality relationship in (2) and define a prior for (1 − *p*_1_)/*p*_1_. The reparameterization becomes a virtual constraint for the common-reference ratios. This is because the increase of the other ratios, as shown in (2), would result in the shrinkage of *R*_2_ (given *p*_1_).

The prior for (1 − *p*_1_)/*p*_1_ was obtained by fitting a model with monoisotopic mass *m* as a covariate to the isotopic distributions of the NCBI data set. A linear relationship between the logarithm of *p*_1_ and *m*, which can be expressed as *p*_1_ = *α* + *βm* + *ɛ*, was found. It can then be transformed to the log-odds scale of *p*_1_. After the transformation, the residuals were observed to be more homoscedastic. Thus, the model takes the form

(3) $$\begin{array}{c} \log\;\left(\frac{1-p_{1}}{p_{1}}\right)=\log\;\left[1-\exp\;\left(\alpha +\beta m\right)\right]-\left(\alpha +\beta m\right)+ɛ, \end{array}$$

where *ɛ* ~ *N*(0, *σ*_*p*_1__^2^). The resulting prior for the common-reference (isotopic) ratio *R*_*l*_*q*__ is lognormal, that is,

(4) $$\begin{array}{cc} & R_{l_{q}}\sim \text{Log-normal}\left(\overset{l}{\underset{i=1}{\sum }}\mu_{i},\overset{l}{\underset{i=1}{\sum }}\sigma_{i}^{2}\right),\\ & \;\;\;\;\;\;\text{where}\;l=3,\ldots ,L, \end{array}$$

and *R*_2_*q*__ = (1 − *p*_1_)/*p*_1_ − ∑_*l*=3_^*L*^ *R*_*l*_*q*__. The prior for (1 − *p*_1_)/*p*_1_ can be obtained from the estimates of the model shown in (3). More specifically,

(5) $$\begin{array}{cc} \frac{\left(1-p_{1}\right)}{p_{1}}\sim \text{Log-normal}\left(\mu_{p_{1}},\sigma_{p_{1}}^{2}\right), & \\ \text{where}\;\mu_{p_{1}}=\log\;\left[1-\exp\;\left(\alpha +\beta m\right)\right]-\left(\alpha +\beta m\right). & \end{array}$$

## 4. Bayesian Model Formulation

In this section, we consider a model for the peak-shape representation of a mass spectrum. 

### 4.1. Model Formulation

We assume that the number of the overlapping peptides, *Q*, is known. Essentially, the model formulation is based on the definition in Section 2. For the observed intensity *y*_*j*_  (*j* = 1,…, *N*), we assume the following model:

(6) $$\begin{array}{c} y_{j}\sim N\left(E\left(y_{j}\right),\sigma^{2}\right), \end{array}$$

with

(7) $$\begin{array}{cc} E\left(y_{j}\right) & =f\left(H,R,M,\sigma_{s}^{2},S\right)\\ & =\overset{Q}{\underset{q=1}{\sum }}\;\overset{L}{\underset{l=1}{\sum }}H_{q}R_{l_{q}}\psi \left(x_{j};M_{q}+\left(l-1\right)S,\sigma_{s}^{2}\right), \end{array}$$

where *x*_*j*_ is the mass coordinate corresponding to intensity *y*_*j*_, **M** = (*M*_1_, *M*_1_,…, *M*_*Q*_) is a vector of monoisotopic masses of the *Q* overlapping peptides, and *S* is the difference in mass locations between two neighboring isotopic peaks of the same peptide, assumed to be constant over all the isotopic peaks for all the overlapping peptides. In (7), *H*_*q*_ is the abundance of the *q*th overlapping peptide (*q* = 1,2,…, *Q*) and **H** = (*H*_1_,…, *H*_*Q*_). Parameter *R*_*l*_*q*__ is the *l*th common reference isotopic ratio for the *q*th peptide, and **R** = (*R*_1_1__, *R*_2_1__,…, *R*_*L*_1__; *R*_1_2__, *R*_2_2__,…, *R*_*L*_2__; …;*R*_1_*Q*__, *R*_2_*Q*__,…, *R*_*L*_*Q*__) is a vector containing the isotopic ratios for all peptides. The function *ψ*(*x*; *μ*, *σ*_*s*_^2^) is a function of a chosen distribution, defined for the shape of the peaks. In this respect, either the difference of a cdf (cumulative distribution function) between two neighboring mass coordinates or a pdf (probability distribution function) can be used. To approximate the (underlying) continuous mass coordinate, we chose to use the cdf, which is also believed to be a more accurate approximation of area under the curve especially when the dispersion parameter *σ*_*s*_ takes very small values. For a normal distribution function, the area under the curve between two neighboring mass coordinates is

(8) $$\begin{array}{cc} & \psi \left(x_{j};M_{q}+\left(l-1\right)S,\sigma_{s}^{2}\right)\\ & \;=\{\begin{array}{c} \Phi \left(x_{j}\;|\;M_{q}+\left(l-1\right)S,\sigma_{s}^{2}\;\right)-\Phi \left(x_{j-1}\;|\;M_{q}+\left(l-1\right)S,\sigma_{s}^{2}\right)\\ \;\;\;\;\;\;\;\;\;\;\;\;\;\;\;\;\;\;\text{if}\;j\geq 2,\\ \Phi \left(x_{j}\;|\;M_{q}+\left(l-1\right)S,\sigma_{s}^{2}\right)\;-\Phi \left(0\;|\;M_{q}+\left(l-1\right)\;S,\sigma_{s}^{2}\right)\\ \;\;\;\;\;\;\;\;\;\;\;\;\;\;\;\;\text{if}\;j=1, \end{array} \end{array}$$

with Φ(*x*_*j*_ | *M*_*q*_ + (*l* − 1)*S*, *σ*_*s*_^2^) denoting the value of the normal cdf function with mean *M*_*q*_ + (*l* − 1)*S* and variance *σ*_*s*_^2^, calculated at *x*_*j*_.

Peaks in MS data often exhibit a right-skewed shape. Thus, an alternative is to approximate the shape by a function that accounts for an asymmetric shape. Asymmetric Laplace function can serve for this purpose. In this case, an extra shape parameter—the skewness parameter *κ*—should be included. The shape function takes the following form:

(9) $$\begin{array}{cc} & \psi \left(x_{j};M_{q}+\left(l-1\right)S,\sigma_{s},\kappa \right)\\ & \;=\{\begin{array}{c} F\left(x_{j}\;|\;M_{q}+\left(l-1\right)S,\sigma_{s},\kappa \right)\\ -F\left(x_{j-1}\;|\;M_{q}+\left(l-1\right)S,\sigma_{s},\kappa \right)\\ \;\;\;\;\;\;\;\;\;\text{if}\;j\geq 2,\\ F\left(x_{j}\;|\;M_{q}+\left(l-1\right)\;S,\sigma_{s},\kappa \right)\\ -F\left(0\;|\;M_{q}+\left(l-1\right)S,\sigma_{s},\kappa \right)\;\\ \;\;\;\;\;\;\;\;\text{if}\;j=1, \end{array} \end{array}$$

with *F*(*x*_*j*_ | *M*_*q*_ + (*l* − 1)*S*, *σ*_*s*_, *κ*) denoting the value of cdf function of an asymmetric Laplace distribution with mean *M*_*q*_ + (*l* − 1)*S* and standard deviation *σ*_*s*_, calculated at *x*_*j*_, that is,

(10) $$\begin{array}{cc} & F\left(x_{j}\;|\;M_{q}+\left(l-1\right)\;S,\sigma_{s},\kappa \right)\\ & \;=\{\begin{array}{c} \frac{\kappa^{2}}{1+\kappa^{2}}\exp\;\left[-\frac{\sqrt{2}}{\sigma_{s}\kappa }\left|x_{j}-\left(M_{q}+\left(l-1\right)S\right)\right|\right]\\ \;\;\;\;\;\;\;\;\;\;\text{if}\;x_{j}<M_{q}+\left(l-1\right)S,\\ 1-\frac{1}{1+\kappa^{2}}\exp\;\left[-\frac{\sqrt{2}\kappa }{\sigma_{s}}\left|x_{j}-\left(M_{q}+\left(l-1\right)S\right)\right|\right]\\ \;\;\;\;\;\;\;\;\;\;\;\;\;\text{if}\;x_{j}\geq M_{q}+\left(l-1\right)S. \end{array} \end{array}$$

### 4.2. Prior Distributions

For *H*_*q*_, *σ* ^2^, *σ*_*s*_, *S*, and *κ*, we use the following noninformative or weak-informative priors:

(11) $$\begin{array}{c} H_{q}\sim N\left(0,\frac{1}{\tau }\right)I\;\left(H_{q}\geq 0\right)\;\;\text{with}\;\tau \sim \Gamma \left(\alpha^{*},\beta^{*}\right),\\ \sigma^{-2}\sim \Gamma \left(\alpha ,\beta \right),\\ \sigma_{s}\sim N\left(0,10^{6}\right)I\;\left(0\leq \sigma_{s}\leq 0.5\right),\\ S\sim N\left(1,\frac{1}{\tau_{s}}\right)\;\text{with}\;\tau_{s}\sim \Gamma \left(\alpha^{**},\beta^{**}\right)I\;\;\left(\tau_{s}\geq 1600\right),\\ \kappa \sim U\left(0.01,0.99\right), \end{array}$$

where *α*, *β*, α*, β*, α**, and β** are positive constants close to zero. To avoid numerical problems, *H*_*q*_ is constrained to be nonnegative. The peak-width parameter *σ*_*s*_ is constrained to be positive and not larger than 0.5, because peaks observed in a spectrum are clearly separated from each other, with the width of a peak not larger than 1 Da. Parameter *S* reflects the average difference in molecular weight of the isotopes and is usually very close to one. Thus, *S* is constrained to be close to one by setting a lower bound for the precision parameter *τ*_*s*_. The skewness parameter *κ* (for the asymmetric Laplace function) is constrained to be smaller than one since the peak envelops are always right skewed (at least in the MALDI-TOF data).

The informative priors for the isotopic ratios are defined by (4) and (5). 

#### 4.2.1. Bayesian Model Averaging for the Estimation of Monoisotopic Masses *M*

**Figure 3:**
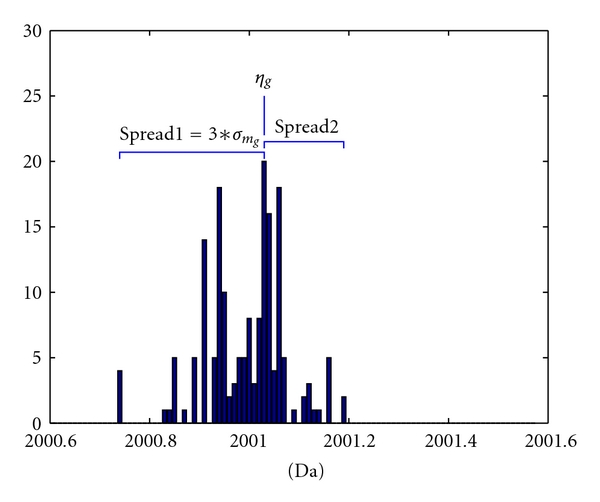
Graphical demonstration of the estimation of mean *η*_*g*_ and standard deviation *σ*_*m*_ for the prior normal density of *M*_*q*_: *η*_*g*_ is chosen to be the mode of the “cluster”; *σ*_*m*_*g*__ is taken as a third of the maximum of *spread*1 (left spread) and *spread*2 (right spread); *σ*_*m*_ is defined as the maximum value of *σ*_*m*_1__,…, *σ*_*m*_*G*__.

Figure 2 shows that monoisotopic masses appear in “clusters” of bell shape. This suggests that a suitable choice for the prior distribution of **M**, at a specific “cluster” for the possible mass range of **M**, may be a normal distribution. Thus, the prior for the monoisotopic mass of the *q*th peptide is defined as follows:

(12) $$\begin{array}{c} M_{q}\sim N\left(\eta_{g},\sigma_{m}^{2}\right), \end{array}$$

where *g* = 1,…, *G*, with *G* being the number of “clusters,” at which the monoisotopic masses are likely to occur. For instance, assuming that the monoisotopic mass of a certain peptide can vary in the mass range of [1997.5,2002.5] Da, as shown in Figure 2, then *G* = 5, as there are five “clusters” shown in the figure. Mean *η*_*g*_ and variance *σ*_*m*_^2^ can be estimated from the NCBI data (as illustrated in Figure 3).

To consider all the *G* possible locations “clusters,” which can possibly contain the true value of the monoisotopic mass of the overlapping peptide, a Bayesian model averaging approach can be considered. More specifically, *G* candidate models are fitted, each with a normal prior *N*(*η*_*g*_, *σ*_*m*_^2^), and *g* = 1,…, *G*. The resulting parameter estimates are a weighted sum of the *G* candidate models. This means that the point estimate of a parameter *θ* is obtained as the weighted average of the model-specific estimates ${\hat{\theta }}_{g}$

(13) $$\begin{array}{c} \hat{\theta }=\overset{G}{\underset{g=1}{\sum }}w_{g}{\hat{\theta }}_{g}, \end{array}$$

where *w*_*g*_ is the weight of the *g*th model. Based on the DIC (deviance information criterion) of each model, *w*_*g*_ can be computed as follows [6]:

(14) $$\begin{array}{c} w_{g}=\frac{\exp\;\left(-\left(1/2\right)\Delta \text{DI}{\text{C}}_{g}\right)}{\left[\sum_{g=1}^{G}\exp\;\left(\left(-1/2\right)\Delta \text{DI}{\text{C}}_{g}\right)\;\;\right]}, \end{array}$$

where ΔDIC_*g*_ = DIC_*g*_ − min _g_(DIC_*g*_). The standard error can be computed as [6, 7]

(15) $$\begin{array}{c} \hat{\sigma }\left(\theta \right)=\overset{G}{\underset{g=1}{\sum }}w_{g}\sqrt{{\hat{\sigma }}_{g}{\left(\theta \right)}^{2}+{\left({\hat{\theta }}_{g}-\hat{\theta }\right)}^{2}}, \end{array}$$

where ${\hat{\theta }}_{g}$ and ${\hat{\sigma }}_{g}\left(\theta \right)$ are, respectively, the point estimate and the standard error for parameter *θ* in the *g*th candidate model. 

### 4.3. Conditional Posterior Distributions

The conditional posterior distributions of *H*_*q*_ and *σ* ^2^ can be obtained analytically. On the other hand, because of nonlinearity, there are no analytical solutions for the conditional posterior distributions for *S*, *κ*, *σ*_*s*_, *M*_*q*_, and *R*_*l*_*q*__. These distributions therefore need to be evaluated by numerical (sampling) methods, for example, a Metropolis-Hasting algorithm with acception-rejection rules. 

## 5. Data analysis

To investigate the performance of the proposed modeling approach, we applied the model to real-life and simulated data. The model was fitted by using the * R* package * R2WinBUGS*, built in * R* to automatically call the * (WinBUGS1.4)* software, which allows to fit Bayesian models. 

### 5.1. Bovine Cytochrome C Mass Spectra

The model was applied to a data set of replicated joint mass spectra obtained for peptides of bovine cytochrome C from LC Packings. Bovine cytochrome C is a relatively small protein related to mitochondria in a cell. It is a chain of 105 amino acids. A peptide mixture of tryptic digested bovine cytochrome C was purchased from LC Packings and mixed with five internal standards from Laser BioLabs used for the calibration of the mass spectrometer. According to the data sheets of the suppliers, the mixture should contain 17 protein fragments. The amino acid sequences and the theoretical monoisotopic masses of these fragments are known.

The peptide mixture was divided into two parts. One part was enzymatically labeled with a stable ^18^O-isotope, with trypsine as a catalyst, while the other part remained unlabeled [8]. In the first case, three units from the unlabeled part were mixed with one unit from the labeled part, which should result in the relative abundance of 1/3. In the second case, three units from the labeled part were mixed with one unit from the unlabeled part, what should result in the relative abundance of 3/1. In both cases, the composed mixture was automatically spotted six times on one stainless steel plate by a robot. The plate was processed by a 4800 MALDI-TOF/TOF analyzer (Applied Biosystems) mass spectrometer and yielded six spectra for the 1/3 mixture and six spectra for the 3/1 mixture.

In the ^18^O labeling strategy, the labeled peptide ideally receives two ^18^O-atoms at its carboxyl terminus, which leads to a four-Da mass shift of the corresponding peptide peaks when analyzed by a mass spectrometer [8]. Thus, each spectrum can be treated as containing pairs (*Q* = 2) of overlapping peptides with the difference in the monoisotopic masses equal to four units of mass difference between two neighboring isotopic peaks, that is, *M*_2_ = *M*_1_ + 4*S* = *M*_1_ + 4 × 1.0015 (see the notation of Section 4).

For the analysis purposes, we chose two peptides with monoisotopic masses of 1456.66 Da and 1584.76 Da. For each peptide, we considered one spectrum for each of two different relative abundances (1/3 or 3/1) of the ^16^O and ^18^O labeled peptides. This results in the following four (sub-)data sets:

Data 1: *M*_1_ = 1456.66248,  *H*_2_/*H*_1_ = 3/1,
Data 2:  *M*_1_ = 1456.66248,   *H*_2_/*H*_1_ = 1/3,
Data 3:  *M*_1_ = 1584.75744,   *H*_2_/*H*_1_ = 3/1,
Data 4:  *M*_1_ = 1584.75744,   *H*_2_/*H*_1_ = 1/3.

**Figure 4:**
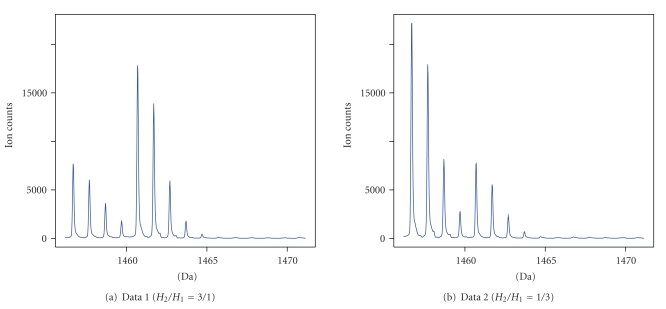
Graphical representation of the first and the second data sets.

A graphical representation of data sets 1 and 2 is shown in Figure 4. 

#### 5.1.1. Results of the Model Fit

Tables 2 and 3 show the means and the standard errors for the parameters of model (6)-(7), based on 100,000 samples, for the four data sets, using asymmetric Laplace function defined by (9)-(10).

The parameters of main interest are

- the estimates of the monoisotopic masses of the two overlapping peptides, *M*_1_ and *M*_2_;
- the relative abundance *H*_2_/*H*_1_.

Note that, usually, instead of the relative abundance, abundances *H*_1_ and *H*_2_ of the overlapping peptides would be of interest. However, in the analyzed experiment, only ratio *H*_2_/*H*_1_ was controlled. Thus, it is of interest to verify whether the proposed models correctly estimate the relative abundance.

In this respect, it is important to mention that, despite the efforts to control the experiment, it appears that, for data sets 1 and 3, the achieved value of relative abundance *H*_2_/*H*_1_ was about 2.4, not 3. The value was estimated by using models for the analysis of ^18^O-labeled mass spectra [9, 10]. This value was therefore assumed as a true relative abundance in Table 2.

Several patterns can be observed from Tables 2 and 3. First, for all of the data sets, the monoisotopic mass of the second peptide *M*_2_ is estimated at the correct peak 5th peak. The monoisotopic mass of the first peptide *M*_1_ is estimated with a negligible bias.

The point estimates of the relative abundance *H*_2_/*H*_1_ are slightly biased downwards. This may be due to the fact that in experiments, in which ^18^O-labeling is used, a part of peptide molecules from a labeled sample do not get a complete label [9, 10]. These incompletely labeled molecules additionally overlap with the molecules from the unlabeled sample. This, in effect, leads to the labeled sample appearing in the spectrum to be less abundant due to the amount of molecules that were incompletely labeled. Thus, the downward bias observed for the estimates of the relative abundance in Tables 2 and 3 may actually reflect this effect.

The point estimates for isotopic ratios **R** are, in general, very close to the true values. Taking the precision measure of the standard errors into account, the differences between these ratio estimates and their true values are negligible. The parameters that describe the shape of the peaks, that is, *S*, *κ*, and *σ*_*s*_, are estimated consistently for different data sets. This indicates that the peak profiles, obtained from the MALDI-TOF experiments, are very similar.

As a comparison, we apply one of the existing approaches, proposed by Breen et al. [3], to the same data sets. It should be noted that by applying this approach, based on the summary statistics, that is, the stick representation for each observed peak, the monoisotopic masses of the two peptides are not estimable. Thus, we merely focus on the comparison of the relative abundance parameter *H*_2_/*H*_1_. The estimated 95% confidence intervals for this parameter for the four data sets are, respectively, (1.9229, 2.5406), (0.2797, 0.3469), (1.9206, 2.5261), and (0.2826, 0.3479). They show severe efficiency loss (as the confidence intervals are much wider) compared with the results presented in Tables 2 and 3. 

### 5.2. A Simulation Study

For illustration purposes and simplicity, the simulation study was based on the model with a normal-density shape function. We considered 30 settings, accounting for various mass differences of the overlapping peptides. The details of the settings are shown in Table 4. Let shift be the integer of the mass difference of the two overlapping peptides, and let tilt be the mass difference after the decimal point. As a result, the mass difference of the two overlapping peptides is equal to *M*_2_ − *M*_1_ = shift + tilt, or in other words, *M*_2_ = *M*_1_ + shift + tilt. It may be difficult to quantify two overlapping peptides when the mass difference between two peptides is too small, that is, either shift or tilt is very small. Thus, it is of interest to investigate different settings with combinations of the two parameters.

In the simulation, we chose three sets of isotopic ratios: an average one (denoted by ** A**), obtained by a Poisson approximation proposed by Breen et al. [3]; the extremely small ratios (denoted by ** E1**); the extremely large ratios (denoted by ** E2**) within 20001 ± 0.5 Da mass range. Sets ** E1** and ** E2** are the isotopic distributions with the second isotopic variant, *p*_2_, being the least and most abundant among all the peptides around 2001 Da from the NCBI data.

The other parameters were chosen as follows:

(16) $$\begin{array}{c} M_{1}=2000.90,\;\;H_{1}=10000,\\ \sigma =10,\;\;\sigma_{s}=0.08,\;\;S=1.0015. \end{array}$$

**Figure 5:**
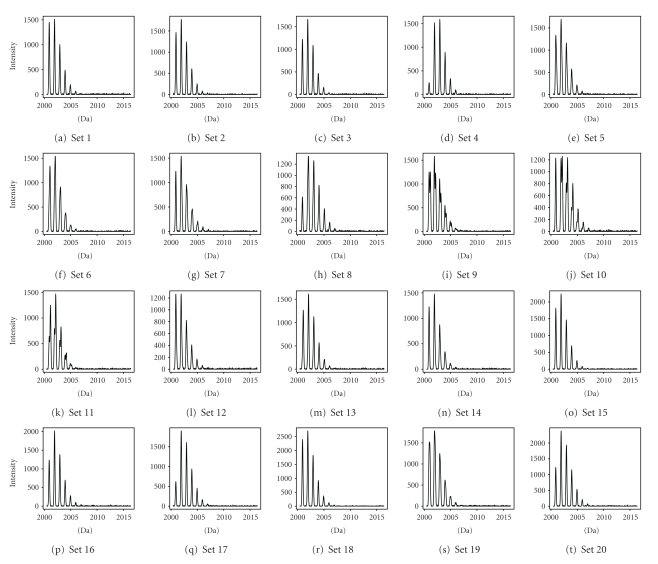
Graphical representation of settings 1–20 of simulated data sets.

**Figure 6:**
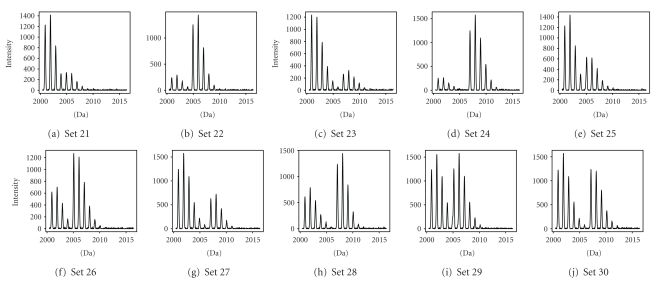
Graphical representation of settings 21–30 of simulated data sets.

For each of the settings, 100 simulated data sets with random noise were generated. Figures 5 and 6 show the graphical representation of the 30 settings. It can be seen that settings 1–3, 5–7, 9–16, 18-19, and 21 are difficult settings, for which the location of the second overlapping peptide is not immediately obvious. In these settings, either the second (overlapping) peptide is much less abundant than the first peptide (e.g., setting 21), or the mass difference between the two peptides is very small (e.g., setting 2).

**Figure 7:**
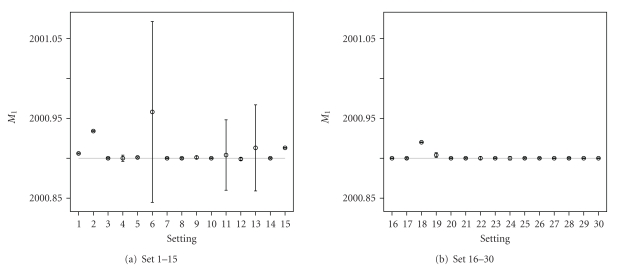
Graphical representation of the average point estimates with the 95% credible intervals for *M*_1_ (true value indicated by the horizontal grey line).

**Figure 8:**
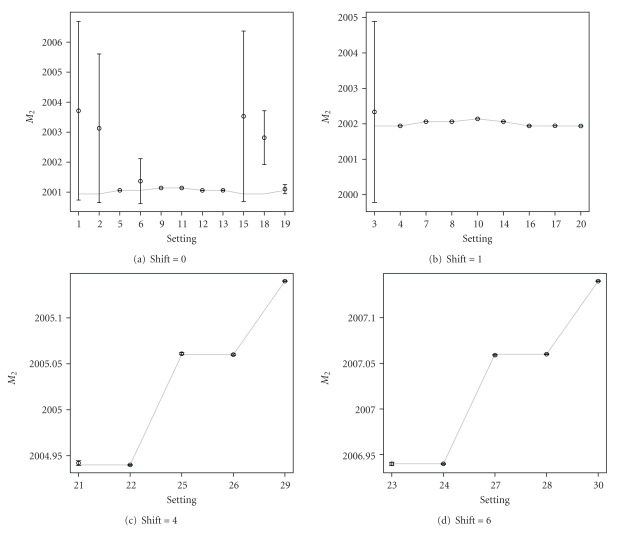
Graphical representation of the average point estimates with the 95% credible intervals for *M*_2_ (true values indicated by the grey line).

**Figure 9:**
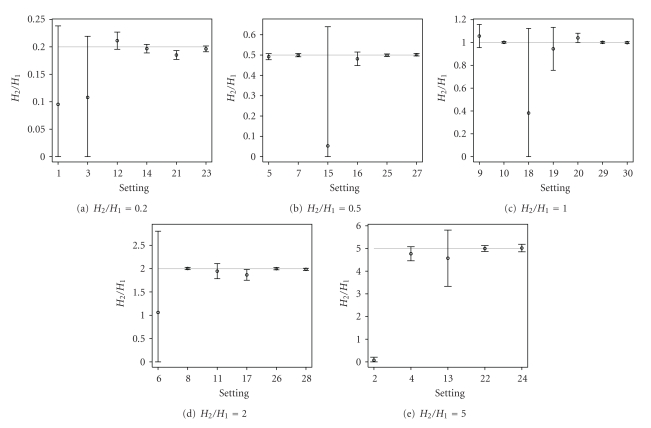
Graphical representation of the average point estimates with the 95% credible intervals for *H*_2_/*H*_1_ (true value indicated by the horizontal grey line).

The graphical representation of the summary statistics for the important parameters is shown in Figures 7–9.  Figure 7 shows, in general, unbiased estimates for parameter *M*_1_, except only for a few of the difficult settings, which exhibit slight bias. The point estimates of the monoisotopic mass for the second overlapping peptide *M*_2_, shown in Figure 8, correctly represent the true mass of the peptide, except only for settings 1–3, 6, 15, and 18. For these settings, the 95% credible intervals, computed based on the model averaging, are very wide. Thus, most of them still contain the true values of *M*_2_. The wide credible intervals are an indication of settings, for which the quantification of the overlapping peptides is difficult. For these difficult settings, the 95% credible intervals for *H*_2_/*H*_1_, as shown in Figure 9, contain zero and thus can be viewed as another indication that the second overlapping peptide is difficult to be found. For the remaining settings, even for some of those, for which the presence of the second peptide is not clear from the data, the Bayesian model averaging approach is able to estimate the monoisotopic masses of the two overlapping peptides and to correctly quantify their relative abundance. A slight bias for the estimation of *H*_2_/*H*_1_ is only observed for setting 21.

**Figure 10:**
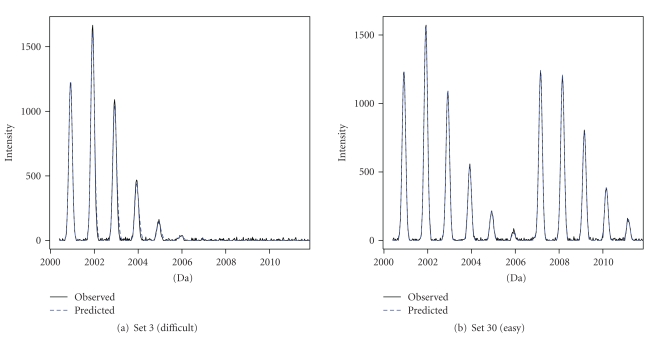
Observed (black solid line) versus predicted (blue-dashed line) spectra (predicted intensity values calculated based on the point estimates of settings 3 and 30).

Figure 10 presents, as an example, the fit of the model to the observed spectra. The figure shows that the fitted spectra correspond to the observed spectra, even for the difficult setting (setting 3).

As can be seen from Table 4, settings 1 to 20 are the settings for which the monoisotopic mass difference of the two overlapping peptides is at most around one Da, that is, shift = 0 or 1. These settings can be viewed as the more difficult ones regarding their relatively small difference in the monoisotopic mass, that is, *M*_2_ − *M*_1_.  Table 5 gives a summary of whether or not the model is able to produce correct estimates (+ indicating correct estimation and − indicating wrong estimation) for these settings, based on the simulation study. Note that poor estimates are produced when the mass difference *M*_2_ − *M*_1_ or the relative abundance *H*_2_/*H*_1_ is too small. In particular, Table 5 indicates that, in general, when *M*_2_ − *M*_1_ ≥ 0.16, the model produces the correct parameter estimates. 

#### 5.2.1. Misspecification of the Number of Overlapping Peptides

In order to investigate the potential influence of the misspecification of the assumed number of overlapping peptides on the parameter estimates, the simulation was repeated for settings 10 and 26, by assuming 3 overlapping peptides (one more than the actual number). The estimates of the first two peptides (ordered according to the estimated masses) were quite similar to the ones obtained by assuming the correct number of overlapping peptides. The abundance parameter for the third peptide was estimated only between 0.07% and 3.66% of the abundance of the second peptide. This indicates that a third peptide may be non-existent and is very likely incurred merely by noise. The BIC (Bayesian information criterion) of the models with two and three overlapping peptides confirmed the nonexistence of the third overlapping peptides. For settings 10 and 26, the BIC for the model with two overlapping peptides were both smaller (7196.5 and 6567.6, resp.) than for the model with three peptides (7259.8 and 6602.1, resp.).

Hence, the two simulation studies show that our modeling approach is robust to the misspecification of the number of overlapping peptides. Moreover, the BIC for models with different number of overlapping peptides gives an indication of the correct number of overlapping peptides, present in the data. 

## 6. Discussion and Conclusions

 The quantification of the overlapping peptides is a difficult problem, because there is often a limited amount of information available in the MS data. A possible solution is to use prior information that could increase the information content of the data. For this reason, in this paper, we have considered the use of a Bayesian approach to analyze high-resolution mass spectra with overlapping peptides. As compared with the existing methods [1, 2], our modeling approach allows for the incorporation of prior information, which should lead to more precise estimates. Moreover, it avoids a multistage analysis, which poses a difficulty in, for example, estimating precision of the obtained estimates.

We have presented the model for the shape representation of a mass spectrum with overlapping peptides, fitted by using the Bayesian model averaging approach. We have investigated the performance of the model with applications to real-life data sets and a simulation study.

The application to the real-life data yielded, in general, estimates corresponding to the true parameter values. The estimates of the relative abundance exhibited a downward bias. This may be due to incomplete labeling of peptide molecules [9, 10]. The modeling approach was compared with one of the existing approaches, proposed by Breen et al. [3], which showed efficiency loss for the parameter of interest. The inefficiency of the parameter estimates can bring about diagnostic problems, by causing false negatives, when applied to clinical diagnostics.

In the simulation study, when applying the modeling approach, we observed, in general, unbiased estimation for the parameters, with either clear or unclear separation for the overlapping peptides in the simulated MS data. Moreover, for the settings, for which the quantification of the second overlapping peptide was difficult, 95% credible intervals of the parameter estimates were wider and contained mostly the true values. This indicates that the width of the 95% credible intervals correctly quantifies the uncertainty of the parameter estimates. Two extra simulations were performed and showed the robustness of the model to the misspecification of the number of overlapping peptides.

The feasibility of the quantification of overlapping peptides depends on the mass difference of the peptides. When Bayesian model averaging approach is applied, it produces unbiased estimates for the parameters related to the overlapping peptides, when the monoisotopic mass difference is at least 0.16 Da, which is roughly a half of the width of an isotopic peak, observed in an MALDI-TOF mass spectrum. This indicates that the two overlapping peptides can be correctly quantified by using the Bayesian model averaging approach when the mass difference of the two peptides is at least a half of the width of an isotopic peak. A smaller mass difference, that is, less than a half of the isotopic peak width, would suggest a complete overlap of the peptides and would make the quantification infeasible.

In summary, the proposed modeling approach offers two advantages:

- it produces unbiased estimates for all settings that show clear or unclear separation of the overlapping peptides in the MS data;
- the model uncertainty, measured by the 95% credible intervals of the parameters, gives an indication of the separability of the overlapping peptides.

Although the method is focused on the application of singly charged MALDI-TOF mass spectrum, it can be modified to apply also for, for example, the doubly charged mass spectrum with the modification of the expression for the mean structure of the model and the prior distributions for the corresponding parameters. Moreover, the proposed modeling approach, assuming unknown masses (sequences) of the overlapping peptides, can be modified for the application, in which the masses are known. In such case, the masses of the peptides in the model can be fixed with known values and the model simplifies.

It should be noted that the validity of this approach is based on a proper-preprocessing procedure (for details of preprocessing, refer to Vaikenborg et al. [11]). More specifically, it assumes that a cluster of peptide peaks is correctly found after noise filtering. This implies that, if a part of the isotopic peaks of a cluster is treated as noise generated and discarded, the method would yield biased estimation.

It is also worth noting that, in the analysis, the number of overlapping peptides was assumed to be known. Usually, in practice, this also needs to be estimated. Such estimation may be difficult by using a Bayesian approach since little prior information can assist the analysis. Identifying the number of overlapping peptides can be viewed as a problem of identifying the number of components of a mixture of distributions by applying a likelihood-based testing approach, or by performing a forward model selection approach. The latter approach can be done by fitting models with sequentially increasing number of components, and then by selecting the model which shows the best fit, depending on, for example, the Bayesian information criterion. The feasibility of such an approach was observed from the simulation studies. To check its validity in real applications may require a larger-scaled analysis based on real-life data. This topic will be addressed in future research. 

## Figures and Tables

**Table 1:** The polynomial model coefficient estimates.

	log *C*_2_	log *C*_3_	log *C*_4_	log *C*_5_	log *C*_6_	log *C*_7_	log *C*_8_
*β*_0_	−2.5835	−2.6283	−2.9429	−3.1161	−3.2939	−3.4508	−3.6021
*β*_1_	3.2954	2.3416	2.4265	2.3733	2.4299	2.4994	2.5967
*β*_2_	−1.7098	−1.0856	−1.2003	−1.1854	−1.2464	−1.3110	−1.3932
*β*_3_	0.4594	0.2772	0.3197	0.3176	0.3386	0.3600	0.3865
*β*_4_	−0.0466	−0.0274	−0.0324	−0.0323	−0.0347	−0.0372	−0.0401
*σ* ^2^	0.0035	0.0008	0.0006	0.0010	0.0012	0.0016	0.0019

**Table 2:** Means and standard errors based on model averaging for the parameters of the model with asymmetric Laplace shape function.

Parameter	Data set 1			Data set 3		
	True	Mean	S.E.	True	Mean	S.E.
*R*_2_1__	0.7933	0.7615	0.01930	0.8703	0.8419	0.01850
*R*_3_1__	0.3567	0.4357	0.01472	0.4223	0.5305	0.01507
*R*_4_1__	0.1166	0.1536	0.009825	0.1478	0.1973	0.01213
*R*_5_1__	0.0306	0.0324	0.002528	0.0413	0.0431	0.003362
*M*_1_*	1456.66	1456.668	0.0012	1584.76	1584.762	0.0007

*R*_2_2__	0.7933	0.7717	0.008791	0.8703	0.8571	0.008971
*R*_3_2__	0.3567	0.3362	0.006908	0.4223	0.4057	0.007161
*R*_4_2__	0.1166	0.1104	0.005162	0.1478	0.1417	0.005569
*R*_5_2__	0.0306	0.0315	0.002299	0.0413	0.0419	0.002860
*M*_2_*	1460.67	1460.676	0.0009	1588.77	1588.771	0.0005

*σ*	—	232.6181	6.6199	—	217.1335	6.5086
*σ*_*s*_	—	0.0734	0.0007	—	0.0770	0.0007
*κ*	—	0.8637	0.01402	—	0.8095	0.007834
*S*	—	1.0018	0.0006	—	1.0029	0.0004
*H*_2_/*H*_1_	2.4	2.2519	0.03791	2.4	2.2181	0.03462

**Table 3:** Means and standard errors based on model averaging for the parameters of the model with asymmetric Laplace shape function.

Parameter	Data set 2			Data set 4		
	True	Mean	S.E.	True	Mean	S.E.
*R*_2_1__	0.7933	0.7907	0.008395	0.8703	0.8598	0.008567
*R*_3_1__	0.3567	0.3593	0.006669	0.4223	0.4347	0.006836
*R*_4_1__	0.1166	0.1229	0.005162	0.1478	0.1575	0.005743
*R*_5_1__	0.0306	0.0328	0.002562	0.0413	0.0441	0.003436
*M*_1_*	1456.66	1456.674	0.0006	1584.76	1584.764	0.0006

*R*_2_2__	0.7933	0.7842	0.02669	0.8703	0.8487	0.02809
*R*_3_2__	0.3567	0.3508	0.01589	0.4223	0.4072	0.01712
*R*_4_2__	0.1166	0.1202	0.007787	0.1478	0.1493	0.009163
*R*_5_2__	0.0306	0.0322	0.002467	0.0413	0.0430	0.003287
*M*_2_*	1460.67	1460.682	0.0011	1588.77	1588.773	0.0011

*σ*	—	278.7780	7.7737	—	273.1138	7.8657
*σ*_*s*_	—	0.0742	0.0007	—	0.0767	0.0007
*κ*	—	0.8662	0.01141	—	0.7591	0.008672
*S*	—	1.0022	0.0006	—	1.0028	0.0005
*H*_2_/*H*_1_	1/3	0.3059	0.007047	1/3	0.3064	0.007222

**Table 4:** The combinations of parameters used for the 30 settings of the simulation study.

	Set 1	Set 2	Set 3	Set 4	Set 5	Set 6	Set 7	Set 8
*shift*	0	0	1	1	0	0	1	1
*tilt *	0.04	0.04	0.04	0.04	0.16	0.16	0.16	0.16
*H*_2_/*H*_1_	0.2	5	0.2	5	0.5	2	0.5	2
Isotopic ratios	** E1E2**	** E1E2**	** AA**	** AA**	** E2A**	** E2A**	** AE1**	** AE1**

	Set 9	Set 10	Set 11	Set 12	Set 13	Set 14	Set 15	Set 16

*shift *	0	1	0	0	0	1	0	1
*tilt *	0.24	0.24	0.24	0.16	0.16	0.16	0.04	0.04
*H*_2_/*H*_1_	1	1	2	0.2	5	0.2	0.5	0.5
Isotopic ratios	** E2E1**	** E1E1**	** E2A**	** E1E2**	** E1E2**	** AA**	** E2A**	** AE1**

	Set 17	Set 18	Set 19	Set 20	Set 21	Set 22	Set 23	Set 24

*shift *	1	0	0	1	4	4	6	6
*tilt *	0.04	0.04	0.16	0.04	0.04	0.04	0.04	0.04
*H*_2_/*H*_1_	2	1	1	1	0.2	5	0.2	5
Isotopic ratios	** AE1**	** E2E1**	** E2E1**	** E1E1**	** AA**	** AA**	** E1E2**	** E1E2**

	Set 25	Set 26	Set 27	Set 28	Set 29	Set 30		

* shift *	4	4	6	6	4	6		
*tilt *	0.16	0.16	0.16	0.16	0.24	0.24		
*H*_2_/*H*_1_	0.5	2	0.5	2	1	1		
Isotopic ratios	** AE1**	** AE1**	** E2A**	** E2A**	** E2E2**	** E2E1**		

**Table 5:** Estimability for settings with various combinations of *shift*, *tilt,* and *H*_2_/*H*_1_. (+: correct estimation; −: wrong estimation).

*R*_*H*_	1		2				5			
*H*_2_/*H*_1_	*R*_*H*_	*R*_*H*_	1/*R*_*H*_	*R*_*H*_	1/*R*_*H*_	*R*_*H*_	1/*R*_*H*_	*R*_*H*_	1/*R*_*H*_	*R*_*H*_

*shift *	0	1	0	0	1	1	0	0	1	1

*tilt* = 0.04	−	+	−	−	+	+	−	−	−	+
*tilt* = 0.16	+	+	+	−	+	+	+	+	+	+
*tilt* = 0.24	+	+	+	+	+	+	+	+	+	+
